# Impact of heat treatment on antigen detection in sera of *Angiostrongylus vasorum* infected dogs

**DOI:** 10.1186/s13071-017-2366-3

**Published:** 2017-09-16

**Authors:** Nina Gillis-Germitsch, Manuela Schnyder

**Affiliations:** Institute of Parasitology, Vetsuisse Faculty, University of Zurich, Winterthurerstrasse 266a, 8057 Zürich, Switzerland

**Keywords:** *Angiostrongylus vasorum*, Dog, Serum, Immune complexes, Heat treatment, ELISA, Antigen, Serology

## Abstract

**Background:**

In the last decade serological tests for detection of circulating *Angiostrongylus vasorum* antigen and specific antibodies have been developed and adopted for individual diagnosis and epidemiological studies in dogs. Although confirmed positive at necropsy, antigen detection was not possible in single experimentally, as well as naturally infected dogs, possibly due to immune complex formation. The aim of this study was to evaluate the effect of heat treatment on detection of *A. vasorum* antigen in sera of experimentally (*n* = 21, 119 follow-up sera) and naturally (*n* = 18) infected animals. In addition, sera of dogs showing clinical signs consistent with angiostrongylosis (*n* = 10), of randomly selected dogs (*n* = 58) and of dogs with other parasitic infections (*n* = 15) were evaluated. Sera were subjected to heat treatment at 100 °C after addition of 0.5 M EDTA (dilution 1:5) and tested with ELISAs for detection of circulating *A. vasorum* antigen before and after treatment.

**Results:**

Between 5 and 11 weeks post-inoculation (wpi) the percentage of positive untreated samples (experimentally infected dogs) increased over time from 33.3 to 90%. Single samples were still negative between 12 and 15 wpi. Overall, between 5 and 15 wpi, 50.6% (45/89) of the available samples were seropositive. From 3 to 6 wpi EDTA/heat treatment caused a change in 8/34 (23.5%) of the samples, with most (*n* = 6, 17.6%) converting from positive to negative. In contrast, from 7 to 10 wpi, treatment induced a change in 19/52 (36.5%) samples, with all but one converting from negative to positive. Thirteen of 18 naturally infected dogs were antigen positive before and 15 after EDTA/heat treatment, respectively. Untreated samples of 3 dogs with suspected angiostrongylosis were antigen positive, of which only one remained positive after EDTA/heat treatment. One of 58 untreated random samples was antigen positive; this sample became negative after treatment, while another turned positive. One of 15 dogs infected with other parasites than *A. vasorum* was positive before but negative after treatment.

**Conclusion:**

Although heat treatment improves *A. vasorum* antigen detection between 7 and 10 wpi by immune complex disruption, we do not recommend systematic pretreating sera because of reduced antigen detection between 3 and 6 wpi and impairment of antibody detection, if performed contemporaneously.

## Background

*Angiostrongylus vasorum* has become a regularly diagnosed parasite in dogs in many European countries over the last few decades. Due to the manifestation of severe clinical signs, a reliable and efficient method for diagnosing the infection is essential. The frequently used copromicroscopic method, the Baermann-Wetzel technique [1] detecting first stage larvae (L1), has recently been complemented by other techniques, such as enzyme-linked immunosorbent assays (ELISAs) [2, 3] and biomolecular methods [4], as well as by a rapid in-clinic assay (Angio Detect™ Test, IDEXX Laboratories, Westbrook, Maine, USA). The ELISA for detection of circulating *A. vasorum* antigen and the ELISA for detection of specific antibodies, both using monoclonal antibodies, give consistent results over the duration of infection [2, 3, 5]. Antigen can be detected as early as 35 days post-inoculation, however, in some dogs antigen is detected later or, in single cases, not detected at all, although such dogs were shown harboring up to 165 adult parasites [2]. Similar difficulties have been reported for other serological tests detecting parasitic antigen, e.g. in the case of *Dirofilaria immitis* in cats [6]. Little et al. [7] recently reported that heat treatment of sera improves the detection of *D. immitis* antigen in infected cats. The same treatment method was also effectively used for sera of *D. immitis* infected dogs [8, 9]. Comparable heat treatment methods for sensitivity improvement have additionally been reported in the past for sera containing antigens of other pathogens such as *Histoplasma* [10], *Coccidioides* [11], *Aspergillus* [12], *Candida albicans* [13] and human immunodeficiency virus type 1 [14]. Apart from heat treatment, acid dissociation is another method described to improve antigen detection [15–17]. Heat treatment and acid dissociation are both believed to disrupt immune complexes such as antigen-antibody complexes and therefore make antigen accessible again for detection by ELISA [18]. Antigen-antibody complexes were described to occur in infections with different pathogens in dogs, such as with ehrlichiosis [19] or leishmaniosis [17]. They may form if antigen and antibodies are both circulating in a high concentration, thereby masking an infection [20]. Reports for immune complex formation in dogs infected with *A. vasorum* and their pathogenic effect are scant [21]. The aim of this study was to evaluate the effect of heat treatment of sera on antigen detection by ELISA in dogs infected with *A. vasorum*.

## Methods

### Source of sera samples

A total of 220 dog sera were evaluated. One-hundred-and-nineteen sera samples originated from 21 dogs experimentally infected with *A. vasorum* from previously performed studies [22–24] before and at various stages of infection. From eight dogs, weekly samples were available starting before or shortly after inoculation until necropsy. From the other 13 dogs a selected number of sera samples were available. Worm burdens were determined at necropsy (varying between 1 and 170 per animal).

Eighteen sera samples originated from dogs naturally infected with *A. vasorum*, presented between the years 2005 and 2017, confirmed positive with either the Baermann technique or at necropsy.

Ten samples were obtained from dogs with suspected angiostrongylosis showing clinical signs which included one or several of the following: respiratory signs, coagulation disorders, cardiac disease, fever and weakness, but were negative by Baermann analysis.

Fifty-eight additional samples were randomly selected from Swiss dogs presented to a veterinary clinic or practice for different reasons.

Fifteen samples from dogs infected with other parasites than *A. vasorum* were tested for determination of specificity. Samples from dogs infected with *Crenosoma vulpis* (*n* = 1), *Dirofilaria immitis* (*n* = 2), *Dirofilaria repens* (*n* = 3), *Capillaria aerophila* (syn. *Eucoleus aerophilus*) (*n* = 1), *Leishmania infantum* (*n* = 3), *Babesia canis* (*n* = 2) and *Ancylostoma caninum* (*n* = 3) were evaluated.

### Sera treatment methods and assays

All 220 sera were tested untreated with the ELISA for detection of circulating *A. vasorum* antigen according to Schnyder et al. [2] and with the sandwich-ELISA for detection of specific antibodies using somatic *A. vasorum* antigen purified with mAb 5/5 [3].

Two different heat treatment methods were initially evaluated. First, samples were tested with a modified heat treatment method described by Little et al. [7]; briefly, samples were heat treated in a dry heat block for 5 min at 100 °C and centrifuged for 5 min at 13,000× *g*. Due to almost full coagulation of sera occurring during heat treatment, this method was dismissed due to impracticality. Nevertheless, some samples that contained enough liquid supernatant after heat treatment were tested and compared with the second method, and comparable results were obtained (results not shown). This second method, based on heat treatment after addition of EDTA, was slightly modified from Weil et al. [25]; briefly, 0.5 M EDTA (pH 8.0) in a dilution of 1:5 was added to samples before they were heated in a dry heat block for 5 min at 100 °C and centrifuged for 5 min at 13,000× *g*. Supernatants were tested with the antigen and antibody ELISAs mentioned above. Samples were also tested with addition of EDTA only, prior to heat treatment, in order to exclude changes in ELISAs due to addition of EDTA.

Antigen cut-off levels for untreated and treated sera were separately defined as mean plus three times standard deviation of optical density (OD) values of the 58 random dog samples.

All samples were kept frozen and stored at -20 °C before and after use and treatments.

### Statistical analysis

Statistical analysis was undertaken with Microsoft Windows Excel 2007 and IBM SPSS Statistics 22. A one way repeated measure analysis of variance (ANOVA) was conducted to determine differences after treatment applied on sera of experimentally infected dogs. *P* - values of *P* < 0.05 were considered statistically significant.

## Results

### Antigen detection by ELISA in sera from experimentally infected dogs

Sera of dogs collected before inoculation (*n* = 8) and untreated sera of experimentally infected dogs collected prior to three weeks post-inoculation (wpi) (*n* = 9) were all antigen negative (Table 1). After 3–4 wpi, single dogs (3/13 samples, 23.1%) started to be positive. Between 5 and 11 wpi, the percentage of positive samples increased over time from 33.3 to 90%. Single dogs were still negative between 12 and 15 wpi. Overall, between 5 and 15 wpi, 50.6% (45/89) of the available samples were seropositive. Addition of EDTA only did not alter the outcome of these results.

**Table 1:** Detection of *Angiostrongylus vasorum* circulating antigen by ELISA in untreated and EDTA/heat-treated sera of dogs experimentally infected with *A. vasorum*

Weeks post-inoculation	Tested samples	Untreated, positive sera	EDTA/heated, positive sera	Seroconverted negative (-) or positive (+) samples
	*n*	*n* (%)	*n* (%)	*n*
-1	8	0 (0)	0 (0)	–
1	6	0 (0)	0 (0)	–
2	3	0 (0)	0 (0)	–
3	8	2 (25.0)	0 (0)	-2
4	5	1 (20.0)	0 (0)	-1
5	12	4 (33.3)	3 (25.0)	-2/+1
6	9	2 (22.2)	2 (22.2)	-1/+1
7	15	4 (26.6)	10 (66.6)	-1/+7
8	14	5 (35.7)	11 (78.6)	+6
9	13	8 (61.5)	12 (92.3)	+4
10	10	8 (80.0)	9 (90.0)	+1
11	10	9 (90.0)	9 (90.0)	–
12–15	6	5 (83.3)	5 (83.3)	–
Total	119	48 (40.3)	61 (51.3)	-7/+20
Total (5–15 weeks)	89	45 (50.6)	61 (68.5)	-4/+20

After EDTA/heat treatment, of the 22 samples collected before five wpi, none were positive. First positive samples were obtained starting from five wpi, also with increasing percentages over time. However, seven previously positive samples collected between 3 and 7 wpi became negative and additional 20 samples obtained between 5 and 10 wpi became positive following EDTA/heat treatment. Overall, of the 89 samples collected after five wpi, 61 (68.5%) were antigen positive. Particularly between 7 and 10 wpi additional 34.6% (18/52) became positive.

ANOVA showed that results obtained with EDTA/heat-treated samples were significantly different from untreated samples (*F*_(1,118)_ = 6.55, *P* = 0.012).

### Antigen detection in sera from naturally infected, clinically suspect and randomly selected dogs and from dogs infected with other parasites

**Fig. 1:**
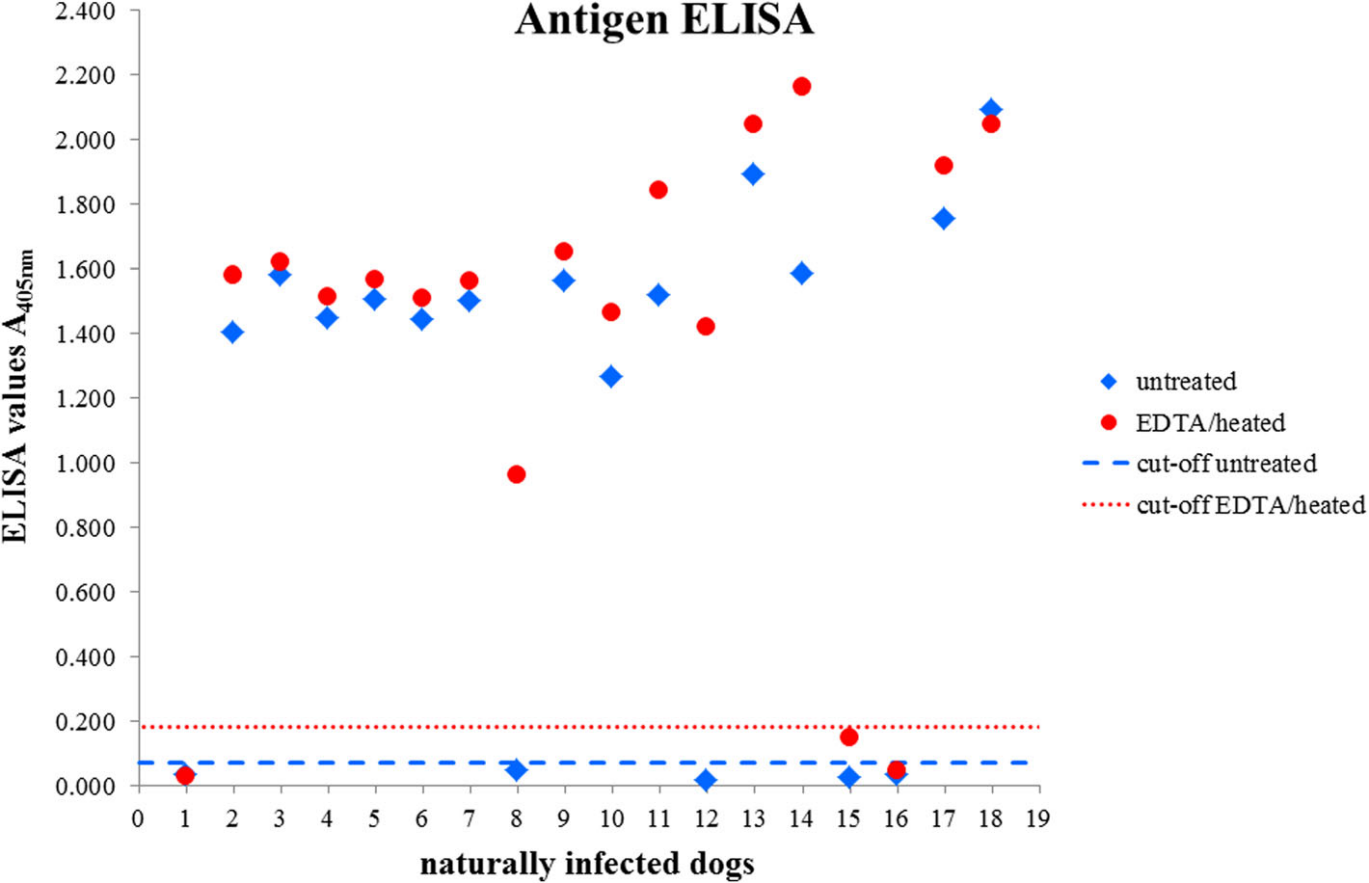
Optical density values of untreated and EDTA/heat-treated serum samples from 18 naturally infected dogs tested for circulating *Angiostrongylus vasorum* antigen

Of the 18 naturally infected positive dogs, 13 were antigen positive prior to EDTA/heat treatment. After EDTA/heat treatment 15 of 18 sera were positive, three samples remained negative (Fig. 1).

Three samples of dogs which were suspected for angiostrongylosis but Baermann negative were initially antigen positive, two of these samples were only marginally above the cut-off. After EDTA/heat treatment, only the one sample which was not marginally above cut-off remained positive, with an increase of the OD value (from 0.887 to 1.252).

**Fig. 2:**
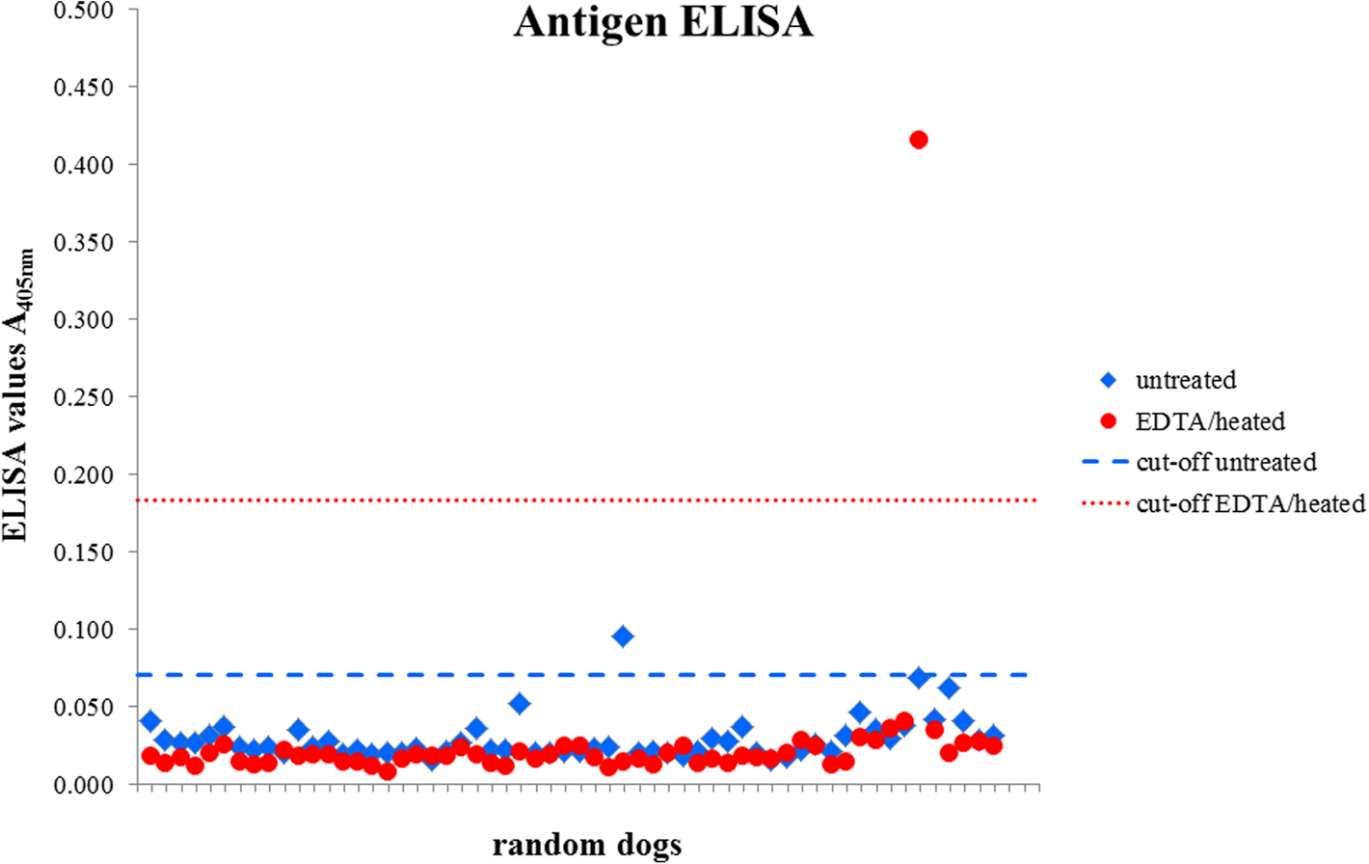
Optical density values of untreated and EDTA/heat-treated serum samples from 58 randomly selected dogs tested for circulating *Angiostrongylus vasorum* antigen

One of 58 random sera samples was antigen positive prior to treatment (slightly above the corresponding cut-off). Addition of EDTA only did not alter these results. After EDTA/heat treatment, the single positive sample became clearly negative, however another sample became antigen positive (Fig. 2).

**Fig. 3:**
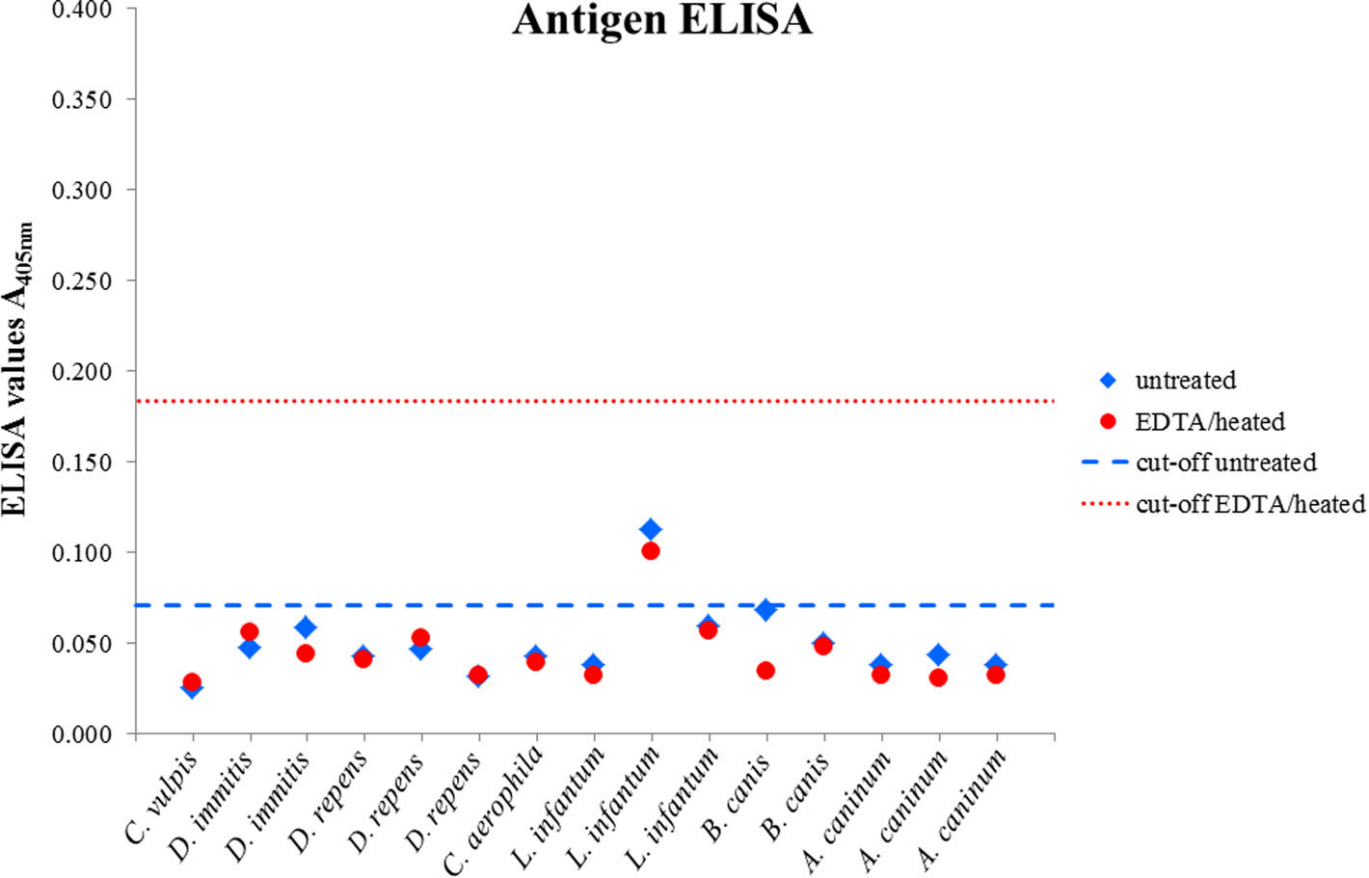
Optical density values of untreated and EDTA/heat-treated serum samples from 15 dogs with proven parasitic infection with *Crenosoma vulpis* (*n* = 1), *Dirofilaria immitis* (*n* = 2), *Dirofilaria repens* (*n* = 3), *Capillaria aerophila* (syn. *Eucoleus aerophilus*) (*n* = 1), *Leishmania infantum* (*n* = 3), *Babesia canis* (*n* = 2) and *Ancylostoma caninum* (*n* = 3) tested for circulating *Angiostrongylus vasorum* antigen

One sample of a dog infected with *L. infantum* was positive before serum heat treatment, with an OD value being only slightly above the cut-off (Fig. 3). After EDTA/heat treatment, this sample was negative. The other 14 samples from dogs infected with other parasites than *A. vasorum* were negative before and after EDTA/heat treatment.

### Antibody detection by ELISA

All samples of experimentally infected dogs collected before six wpi (*n* = 42) were antibody negative, all samples collected after six wpi (*n* = 77) were antibody positive. Of these, after EDTA/heat treatment only 27.3% (21/77) remained positive.

## Discussion

Previous evaluations showed that circulating *A. vasorum* antigen detection in untreated sera is possible starting from five wpi, increasing with duration of the infection, and can reach high sensitivity (95.7%) and specificity (94%) [2]. The presented data with samples obtained under experimental settings confirm these results and show that with heat treatment of sera antigen detection sensitivity by ELISA can be improved between seven and ten wpi: an additional 34.6% of sera became positive. On the other hand, five out of seven previously positive samples collected between three and five wpi turned negative with EDTA/heat treatment, therefore decreasing the sensitivity in this earlier phase of infection, in which clinical signs may also pass unnoticed.

An experimentally infected dog harboring a single adult *A. vasorum* specimen became antigen positive only fifteen wpi (on the day of necropsy) in both the untreated and heat-treated sample, indicating that dogs harboring a small number of adult parasites may have antigen values below the threshold of detection, and that antigen detection cannot be improved by heat treatment in such cases.

More interestingly, single dogs harboring high numbers of parasites were negative in the antigen detection ELISA: three of four untreated samples (collected between three and eight wpi) of one dog harboring 165 adult *A. vasorum* specimens [2] were negative, while all samples of this dog collected after five wpi became positive after heat treatment. In combination with a high worm burden, this could be an indication for hyperimmune sera with blocking antibodies preventing antigen to be detected. Immune complex formation was previously assumed to occur in animals infected with *A. vasorum* [26] and has generally been discussed as the reason for low or absent antigen detection, especially in animals infected with *D. immitis* [8, 27]. Like *A. vasorum*, this filarial nematode has dogs as definitive hosts, also resides in their pulmonary arteries and the heart, and is responsible for potentially fatal infections. Animals infected with *D. immitis* or *A. vasorum* often suffer from persistent chronic infections that can result in hyperglobulinemia, and therefore may induce antigen-antibody complex formation [7, 25, 27–29].

In sera of confirmed naturally infected dogs, antigen detection sensitivity improved, with two sera becoming additionally positive with EDTA/heat treatment. Results obtained with samples of animals with suspected clinical angiostrongylosis (but negative for faecal L1 detection) furthermore suggest that heat treatment may help differentiate between positive and negative samples; two untreated samples that were marginally above the cut-off became negative after EDTA/heat treatment. In contrast, the high OD value of a third dog with signs indicative for angiostrongylosis further increased by heat treatment, suggesting an existing infection. This can indicate that the two samples that became negative were obtained from dogs three to six weeks after infection (and therefore became false negative), and that the dog that remained positive may have been already infected for at least seven weeks (and false negative for coproscopic larval detection).

Heat treatment may cause background or interference, and this could decrease specificity. This was counteracted by adopting a method which is believed not to increase background values [25], and, importantly, by adapting the cut-offs correspondingly. Accordingly, none of the samples from dogs infected with other parasites than *A. vasorum* were positive after heat treatment; one sample of a dog infected with *L. infantum* actually became negative, confirming that there is no loss of specificity. However, we cannot fully exclude that heat treatment may also induce false results in single samples. Among the random sera, a single positive sample being slightly above the corresponding cut-off became clearly negative, while another previously negative became positive with treatment.

Methods other than heating, such as acidification of sera, are described. However, in the case of HIV antigen detection, heat treatment was more effective than acidification [30, 31]. We evaluated heat treatment only and heat treatment with addition of EDTA. Heat treatment only had limitations, because sera tended to almost fully coagulate and therefore there was not sufficient liquid supernatant left for testing for most of the sera. If samples additionally contained EDTA, coagulation did not occur to such extent and more supernatant could be obtained. Thus, this latter method was used and is consequently recommended, but is not advisable for commercially available test kits that use colloidal gold particles, because of the chelating effect of EDTA, or because of other interferences.

A heating technique was recently used by Ciucă et al. [32] to detect *D. immitis* antigen in Romanian stray dogs; detection increased by 18.6% after heat treatment. However, the authors also suggested that heat treatment might have triggered cross-reactions with *D. repens* [32]. Similarly, we cannot exclude that heat treatment may have created interference or background in our tests.

The ELISA for detection of specific antibodies can detect *A. vasorum* antibodies as early as five wpi. The test can be performed simultaneously with antigen detection: together, antigen and antibody testing have the highest positive predictive value and provide indications on ongoing and previous infections for individual dogs and for population studies [5, 33]. In the present study, all untreated sera of experimentally infected dogs collected after six wpi were antibody positive. In contrast, after EDTA/heat treatment only 27.3% (*n* = 21) remained seropositive, indicating that heating antibodies to 75 °C or higher will induce aggregate formation, as previously described [34]. For instance, heating IgGs to 71 °C will lead to denaturation of both F domains [35]. Since our samples were heated to 100 °C it is very likely that detectable antibodies were destroyed through aggregate formation or denaturation.

## Conclusions

Although EDTA/heat treatment improves circulating antigen detection by ELISA for single samples and heat treatment methods are easily performed (if the required laboratory equipment is available), we do not recommend pretreating sera systematically prior to testing. Heat treatment of dog sera improves antigen detection between 7 and 10 wpi, but impairs the detection between 3 and 5 wpi and additionally inhibits antibody detection. Furthermore, controversial results can be observed with randomly selected sera and sera from dogs suggestive of angiostrongylosis in the antigen detection ELISA with heat treatment.

In select cases, i.e. negative antigen results in dogs with typical clinical signs suggestive of angiostrongylosis and (and/or negative coproscopic results), it may be nevertheless an option to pretreat sera. As early diagnosis is essential for appropriate treatment and prevention of complications, repeating the initial testing may be therefore recommended, and will rule out immune complex formation in previously missed *A. vasorum* infections.

## Abbreviations

EDTA: ethylenediaminetetraacetic acid
ELISA: enzyme-linked immunosorbent assay
L1: first stage larvae
OD: optical density
wpi: weeks post-inoculation
